# The central role of biological data mining in connecting diverse disciplines

**DOI:** 10.1186/1756-0381-6-14

**Published:** 2013-08-12

**Authors:** Jason H Moore, Marylyn D Ritchie

**Affiliations:** 1 Departments of Genetics and Community and Family Medicine, Institute for Quantitative Biomedical Sciences, The Geisel School of Medicine, Dartmouth College, One Medical Center Dr., Lebanon, NH 03756, USA; 2 Center for Systems Genomics, Department of Biochemistry and Molecular Biology, The Pennsylvania State University, 503 Wartik Lab, University Park, PA 16802, USA

## 

Scientific research has always suffered from the compartmentalization of different disciplines. This historic phenomenon is beginning to erode in specific broader disciplines such as medicine where new interdisciplinary areas such as bioinformatics have worked to bring together biologists with computer scientists and mathematicians. This is an encouraging trend but has yet to percolate to more disparate disciplines where there is tremendous potential for scientific advances. We had the pleasure and honor of participating in a 2013 Indonesian-American Kavli Frontiers of Science Symposium organized and sponsored jointly by the United States National Academy of Sciences and the Indonesian Academy of Sciences. This invitation-only scientific conference took place in Bali, Indonesia and was attended by young or early career scientists (Kavli Fellows) doing cutting-edge research in extremely diverse disciplines. The goals of the conference were two-fold. The first goal of was to expose researchers to very broad research topics to promote new research ideas within their own field or to expand their research into new fields. The second goal was to stimulate collaboration between U.S. and Indonesian researchers to foster a global exchange of ideas.

The Indonesian-American Kavli Frontiers of Science Symposium consisted of six scientific sessions including social networks, personalized medicine, biodiversity and biogeography, life in extreme environments, renewable energy and synthetic biology. Each of these diverse sessions had three speakers followed by a lengthy panel discussion. Topics covered in each session were timely with global significance. For example, the personalized medicine session focused on topics such as mining public databases to provide insights into drug development while the biodiversity session focused on topics such as developing barcodes for all the organisms in a particular ecosystem. There were also a number of poster presentations from scientists representing each of these areas.

The Kavli Frontiers of Science Symposium series (http://www.nasonline.org/kfos) is the first that we have attended that exposes scientists to such a diverse set of disciplines with the added benefit of global context. Seeing all of these topics collectively allows us as a community to begin thinking about scientific questions that rise above our own narrow research interests. For example, our own research areas are most closely aligned with personalized medicine and the genetics of common human diseases. However, it was easy to begin to see research opportunities that might cross multiple seemingly unrelated disciplines. For example, we firmly believe that genetic variation impacts susceptibility to common diseases such as cancer in the context of our ecology and environmental exposure. It is not too much of a stretch to see how biodiversity might impact human health through the food supply (i.e. nutrition), for example. One can also see the immediate impact of social networks on human health (e.g. biological connections, support networks, etc.). Metagenomics of life in extreme environments is critical to understanding our own microbiome that provides an important environmental layer that greatly influences risk of human disease. Synthetic biology provides a new type of genetic engineering that could provide new drugs and vaccines to treat disease. Finally, it is easy to see how important renewable energies are for replacing carbon-based sources such as oil and coal that produce toxic byproducts that certainly interact with our genomes to influence disease risk. Although some might be skeptical about participating in such a diverse curriculum, it quickly becomes obvious how this diverse community of scientists are intertwined with much to share and learn from each other.

We would like to see more conferences organized across diverse disciplines. We think that experts in biological data mining have a very central role to play in bringing scientists together from different backgrounds and interests. Analytics, artificial intelligence, data mining, data science and machine learning represent a common currency that can be used to bring teams of researchers from all academic areas, especially those with big data. Our future impact on humanity must be bigger than our own narrow research interests and applications.

